# Large language model (ChatGPT) as a support tool for breast tumor board

**DOI:** 10.1038/s41523-023-00557-8

**Published:** 2023-05-30

**Authors:** Vera Sorin, Eyal Klang, Miri Sklair-Levy, Israel Cohen, Douglas B. Zippel, Nora Balint Lahat, Eli Konen, Yiftach Barash

**Affiliations:** 1 Department of Diagnostic Imaging, Chaim Sheba Medical Center, Tel Hashomer, Israel; 2 Sackler School of Medicine, Tel-Aviv University, Tel-Aviv, Israel; 3 DeepVision Lab, Chaim Sheba Medical Center, Tel Hashomer, Israel; 4 Sami Sagol AI Hub, ARC, Chaim Sheba Medical Center, Tel Hashomer, Israel; 5 Department of General and Oncological Surgery- Surgery C, Chaim Sheba Medical Center, Tel Hashomer, Israel; 6 Department of Pathology, Chaim Sheba Medical Center, Tel Hashomer, Israel

**Keywords:** Breast cancer, Cancer imaging

## Abstract

Large language models (LLM) such as ChatGPT have gained public and scientific attention. The aim of this study is to evaluate ChatGPT as a support tool for breast tumor board decisions making. We inserted into ChatGPT-3.5 clinical information of ten consecutive patients presented in a breast tumor board in our institution. We asked the chatbot to recommend management. The results generated by ChatGPT were compared to the final recommendations of the tumor board. They were also graded independently by two senior radiologists. Grading scores were between 1–5 (1 = completely disagree, 5 = completely agree), and in three different categories: summarization, recommendation, and explanation. The mean age was 49.4, 8/10 (80%) of patients had invasive ductal carcinoma, one patient (1/10, 10%) had a ductal carcinoma in-situ and one patient (1/10, 10%) had a phyllodes tumor with atypia. In seven out of ten cases (70%), ChatGPT’s recommendations were similar to the tumor board’s decisions. Mean scores while grading the chatbot’s summarization, recommendation and explanation by the first reviewer were 3.7, 4.3, and 4.6 respectively. Mean values for the second reviewer were 4.3, 4.0, and 4.3, respectively. In this proof-of-concept study, we present initial results on the use of an LLM as a decision support tool in a breast tumor board. Given the significant advancements, it is warranted for clinicians to be familiar with the potential benefits and harms of the technology.

The release of the chatbot ChatGPT by OpenAI has gained a lot of public, media, and scientific attention. GPT (Generative Pre-training Transformer) is a large language model (LLM). LLMs are based on transformer with Attention mechanism^1^. These models are trained on extremely large datasets, consisting of billions of words and parameters. They are also considered “few-shot learners”, meaning that once trained, they can adapt to new domains with a small number of examples. LLMs can be used for various applications in clinical care and research^2^. They can be used as a support tool for physicians, allowing quick summarization of data, question answering, and even treatment suggestions. There are some reports as well as several studies that have shown encouraging results on ChatGPT performance in various complex medical tasks. These include the United States Medical Licensing Exam (USMLE)^3^, imaging reports simplification for patients^4^, decision-making on the appropriate breast imaging examinations^5^, and scientific manuscripts generation^6^.

Medical tumor boards generally present and discuss the most complex clinical cases. The active involvement in tumor boards represents formidable intellectual challenges for oncologists, surgeons, radiologists, and pathologists in their clinical practice. This is due to the need to integrate medical expertise, intricate medical understanding and insightful clinical judgment. In the current proof-of-concept study we aim to evaluate a large language model (ChatGPT-3.5) as a support tool for a breast tumor board.

Ten consecutive women presented in our institutional tumor board were included in this study. The mean age was 49.4. Eight (8/10, 80%) of the patients had invasive ductal carcinoma (IDC), one patient (1/10, 10%) had a DCIS and one patient (1/10, 10%) had a phyllodes tumor with atypia. Patient characteristics are detailed in Table 1.

**Table 1:** Patient characteristics.

Age	Family history	Cancer type	Current clinical status	Size (cm)	Ki67	ER	PR	HER2	LN	Tumor board	ChatGPT-3.5
51	Maternal cousin (29)	Paget	History of multifocal IDC in her left breast with lymph nodes involvement in 2021. Underwent lumpectomy followed by radiation, Tamoxifen and chemotherapy. MRI on 06.12.2022 was normal. In March 2023 had discharge and an ulcer in her left nipple. Punch biopsy result was Paget ER negative.	N/A	N/A	−	NA	NA	NA	Nipple-areola complex excision	“Surgical excision of the affected area”
47	Mother (48), maternal grandmother (48), paternal grandmother.	IDC grade 2	Palpable breast mass, resulted in newly diagnosed IDC, on MRI single tumor and no lymphadenopathy.	1.2	40%	+	−	−	−	Lumpectomy and sentinel	Neoadjuvant endocrine therapy
46	None	DCIS intermediate high grade	Diagnosed with DCIS, underwent lumpectomy and sentinel, lymph nodes were clear. DCIS on pathology was multifocal with involved inferior margin and other margins were close. The patient then completed radiation to the breast and was started on Tamoxifen.	Multi-focal	N/A	+	+	−	−	Re-lumpectomy	Re-excision or radiation boost. Consider a mastectomy if re-excision is not feasible
54	Maternal aunt (70+)	Phyllodes tumor with mild atypia	Underwent biopsy for a palpable mass measuring 6 cm at US, and the biopsy result was phyllodes tumor with mild atypia. The tissue received for pathology analysis was fragmented therefore assessment of surgical margin was not possible.	6	NA	NA	NA	NA	NA	MRI to evaluate post-operative lesion extent	Re-excision
42	None	IDC grade 2	Newly diagnosed IDC with lymph node involvement	1.1	25%	+	+	−	+	Lumpectomy with axillary lymph nodes dissection	Lumpectomy with axillary lymph nodes dissection
63	Grandmother	IDC grade 2	Newly diagnosed IDC, bilateral. No lymphadenopathy. One enhancing suspicious focus at MRI that was not biopsied	1.7, 1	25%, 9%	+	+	+	−	Targeted US to the additional enhancing focus and biopsy if needed. Then consider bilateral lumpectomy.	Neo-adjuvant therapy
33	Paternal grandmother (43)	IDC grade 2	Diagnosed with multifocal IDC, Completed neoadjuvant chemotherapy with Taxol and Herceptin. On follow-up MRI complete radiological response.	multifocal	80%	+	+	+	−	Mastectomy	Mastectomy
40	None	IDC with DCIS	Newly diagnosed IDC + DCIS. On MRI segmental scattered enhancement in the left breast in dimensions 7.5×4 cm involving the nipple and skin. No lymphadenopathy. On additional biopsy from far from the initial mass - high grade DCIS. PET-CT was negative for distant metastases.	7.5	30%	+	+	−	−	Mastectomy	Mastectomy
51	None	IDC grade 3	Newly diagnosed IDC, single mass and no lymphadenopathy at MRI. PET-CT with no evidence of distal metastases.	2	80%	+	−	−	−	Lumpectomy and sentinel	Lumpectomy and sentinel
66	BRCA2 carrier	IDC grade 2	BRCA2 carrier, previously underwent risk-reducing bilateral salpingo-oophrectomy. Diagnosed with IFC.	0.8	20%	+	+	−	−	Lumpectomy and sentinel	Lumpectomy and sentinel. However, given the patient’s age, overall health, and history of bilateral salpingo-oophrectomy, mastectomy should also be discussed.

*IDC* invasive ductal carcinoma, *DCIS* ductal carcinoma in-situ, *LN* lymph nodes.

In seven out of ten cases (7/10, 70%), ChatGPT’s recommendations were similar to the tumor board’s decisions. Based on the first reviewer, mean score for the chatbot’s summarization of the clinical vignettes was 4.6, foragreement with clinical recommendations 3.7, and for explanations 4.3. Mean values for the second reviewer were 4.3 for summarization, 4.0 for agreement with clinical recommendations, and 4.3 for explanations (Fig. 1). Agreement between raters was fair for summarization (k_w_ coefficient = 0.42, 95% CI 0.10–0.50), substantial for clinical recommendation (k_w_ coefficient = 0.80, 95% CI 0.78–0.81), and substantial for explanation (k_w_ coefficient = 0.65, 95% CI 0.53–0.74).

**Fig. 1:**
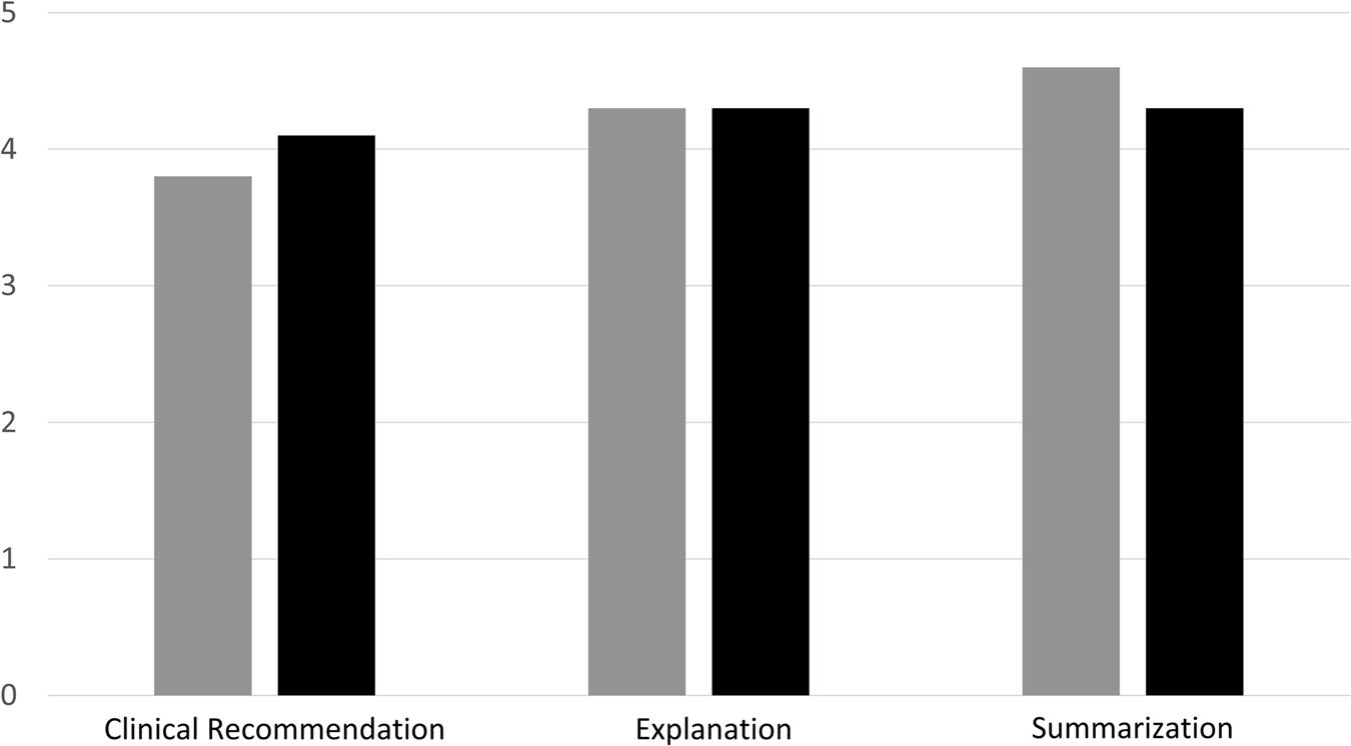
Rating of the performance of a large langue model (ChatGPT) in three categories by the two reviewers (M.S.L. – reviewer-1 in gray and Ey.K. – reviewer-2 in black): summarization of text, clinical recommendation, and explanation on the decision made.

In eight cases (8/10, 80%) the chatbot recommended surgery as the next management step, and in two cases (2/10, 20%) it recommended neoadjuvant chemotherapy treatment. According to the tumor board recommendations, seven patients were referred for surgery, two to imaging and one to neoadjuvant chemotherapy. When recommending a multidisciplinary consult as an additional note in the generated responses, never did ChatGPT mention radiologist as part of the medical forum.

This study evaluates the performance of ChatGPT as a clinical decision support tool in patient management in breast tumor board decisions. Our findings showed that the chatbot’s clinical recommendations were in-line with those of the tumor board in 70% of cases. Furthermore, the chatbot provided concise summaries for the clinical cases, and explanations for its conclusions. Notably, lowest grading scores, from both reviewers, pertained to the chatbot’s clinical recommendations. Performances in summarization and explanation were rated higher. Indeed, deciding on clinical management is the most challenging intellectual task, requiring medical understanding and expertise. It is interesting to note that in one of the cases the chatbot “missed” information on the patient’s HER2 FISH results. However, when asked directly, it corrected the error. We speculate that this may be due to the model’s use of Attention mechanism. Attention is a key aspect of Transformer-based language models like ChatGPT. It allows to analyze the context of words by taking into account surrounding words and weighing them based on relevance. Transformers process all words at the same time and calculate attention weights between them. It may not have put enough weight on the FISH results. This highlights its tendency in some cases to overlook important information, as in our patient and her HER2 status.

Another interesting point is the lack of referral in all cases to additional imaging, or consultation with a radiologist as part of a multidisciplinary team. That is despite the fact that radiologists have important roles in tumor boards while discussing patient management. Radiologists assist in determining the stage of breast cancer and contribute to treatment planning and evaluation of treatment response. Historically, there have been misperception of the general public on radiologists. It is common that patients do not recognize radiologists as one of their treating physicians, or in some cases as physicians at all^7^. As ChatGPT’s training was based on texts from the internet, it may have caused some sort of replication or even amplification of that trend. There are some possible ways by which the model’s recognition of radiologists’ roles can be improved. As language models increase in size and are trained on larger data, they may better comprehend the nuances of radiologists’ responsibilities. Additionally, large language models can be fine-tuned with specific medical or radiological data. Exposure to domain-specific information should enhance the model’s “knowledge” in the field. Finally, it may be possible that the model did not assign sufficient weight to radiologist consultations in the decision-making process. The latter issue can be adjusted and corrected with training. There are several limitations to this study. The proof-of-concept nature of the study limited the sample size to a mere ten patients, which does not reflect the algorithm’s performance in real-world clinical settings. Consequently, generalizing the results from such a small sample size is unfeasible. Furthermore, some tumors and many clinical scenarios are not represented at all. For example, all eight IDC cases were ER-positive. Although ER-positive breast cancer is more common^8^, decisions on triple–negative and HER2-positive patients’ management may be more complicated. Furthermore, there was only one case of DCIS, and no cases of invasive lobular carcinoma (ILC) at all. Finally, one of the reviewers (M.S.L.), participates regularly in the tumor board. Thus, there is a possibility of subjective bias in the grading process.

There are inherent limitations to ChatGPT that must be considered. One concern is the potential generation of false or incorrect information, which may lead to inappropriate medical decisions and compromise patient safety. The output of the chatbot is impacted by the data it is trained on. Thus, if training data do not represent diverse populations, bias may be introduces, and potential exacerbation of healthcare disparities^9^. Moreover, it is important to consider that ChatGPT generates responses based on the dataset it was trained on. The data may not be up-to-date, particularly in fields such as oncology, where new trials and drugs are constantly emerging.

Another issue is the question of legal responsibility and liability in cases where AI-driven decisions lead to negative outcomes. Data security is an additional critical issue. The insertion of actual patient data into these models necessitates data protection mechanisms to prevent unauthorized access. The potential for adversarial cyber-attacks, where malicious actors manipulate the AI system to produce harmful outcomes, is a growing concern^10^. This emphasizes the significance of developing cybersecurity measures to safeguard AI systems in clinical settings.

To conclude, in this study we demonstrate the performance and feasibility of use of ChatGPT-3.5 in one of the most complex tasks in patient care. We identify strengths and discuss pitfalls that still need to be addressed. Further studies with larger sample sizes are warranted in order to establish the actual performance of the chatbot in different clinical scenarios. Given the significant advancements, it is likely that the use of LLMs such as ChatGPT as an assisting and supporting tool for physicians will expand and evolve. Thus, familiarity of clinicians on the pros and cons of this technology is essential.

## Methods

This retrospective study was approved by the Chaim Sheba Medical Center institutional review board (IRB) with a waiver of informed consent granted and so participants didn’t provide written informed consent. Ten consecutive women who were diagnosed with breast cancer and were presented at breast tumor board at our institution in January 2023 were included in the study. Women who underwent imaging outside of our institution were excluded to ensure homogeneity of data.

For each patient, we collected the most recent clinical notes from both oncology and surgery clinic visits, the latest imaging results (including mammography, ultrasound, and MRI), surgical report if available, and the corresponding pathology results, or results from biopsy.

Y.B. extracted the relevant clinical data. V.S. generated clinical vignettes summarizing the relevant clinical data, while blinded to the tumor board decisions. Vignettes included demographic information, clinical history, imaging and pathology results, and current status (whether underwent surgery, borders, molecular tests). The vignettes were written in English. It should be noted that some of the notes and imaging results were originally in Hebrew, and thus were translated.

Y.B. inserted the clinical vignettes into ChatGPT-3.5, and asked the chatbot to recommend on the next most appropriate step in management. We gave it options from which it was supposed to choose. The exact query was: “Hi, can I give you a patient story of breast cancer detected and you’ll say what is the next step in her management? please decide if she needs surgery, what type of surgery, whether she needs neoajuvant therapy before, or does she needs further testing”. ChatGPT-3.5 was accessed on February 9th, 2023, and all answers were obtained at that time. We then retrieved the summary and decision notes from the tumor board, documented regularly in the medical chart of each patient following tumor board discussion. Y.B. and V.S. reviewed the chatbot’s answers and compared them to the tumor board’s decisions, asking the chatbot to elaborate when recommendations were divergent. All cases were then reviewed and discussed together with M.S.L., who is a senior breast radiologist participating regularly in the tumor board.

M.S.L. graded the responses based on three distinct categories: summarization, clinical recommendation, and explanation. A grading scale of 1–5 was used, where 1 indicated complete disagreement, 2 disagreement, 3 neutrality, 4 agreement, and 5 complete agreement (Supplementary Tables 1–3). Clinical recommendations were also evaluated using a binary grading system, focusing on whether the overall recommendations regarding surgery, systemic treatment, and further assessment were aligned between the tumor board and the chatbot.

Due to the possibility of bias introduction since M.S.L. usually participates in the tumor boards, Ey.K. who is a senior radiologist not involved in neither data collection nor in the tumor boards, also graded independently the chatbot’s responses. Agreement between raters was measured using linear weighted Cohen’s kappa (k_w_) coefficient. Interpretation of the k_w_ coefficient was as follows: −0: less than chance agreement; 0.01–0.20: slight agreement; 0.21–0.40: fair agreement; 0.41–0.60: moderate agreement; 0.61–0.80: substantial agreement; 0.81–0.99: almost perfect agreement^11^.

### Reporting summary

Further information on research design is available in the Nature Research Reporting Summary linked to this article.

## Supplementary information

Supplemental

reporting summary

## Data Availability

The data that support the findings of this study are available upon request from the corresponding author [V.S.].
