# Supplementary material for: Large language model (ChatGPT) as a support tool for breast tumor board
Source: NPJ Breast Cancer. 2023 May 30;9:44. doi: 10.1038/s41523-023-00557-8 (PMC10229606; doi:10.1038/s41523-023-00557-8)
Supplement: Supplementary file 1 — Supplemental [file 41523_2023_557_MOESM1_ESM.docx]

**Supplemental**

**Grading Scales:**

**Supplementary Table 1. Summarization:**

| Grade | Definition |
| --- | --- |
| 1 - Poor (Total Disagreement) | The algorithm-generated summary fails to capture the essential information from the clinical data. It may contain inaccuracies or irrelevant information. |
| 2 - Below Average (Disagreement) | The summary captures some important points from the clinical data, but misses other key aspects. It may contain minor inaccuracies or redundancies. |
| 3 - Average (Neutrality) | The summary adequately represents the key points of the clinical data. It is generally correct and comprehensible, but could be improved in terms of clarity or conciseness. |
| 4 - Above Average (Agreement) | The summary effectively captures the important information from the clinical data. It is accurate and clear, requiring minimal improvements. |
| 5 – Excellent (Total Agreement) | The algorithm-generated summary perfectly captures the essential information from the clinical data. It is highly accurate, clear, and concise, leaving no room for improvement. |

**Supplementary Table 2. Clinical Recommendation Scale:**

| Grade | Definition |
| --- | --- |
| 1 - Poor (Total Disagreement) | The algorithm's treatment recommendation is entirely incorrect or potentially harmful for the patient. It may have misinterpreted or ignored critical clinical data in the decision-making process. |
| 2 - Below Average (Disagreement) | The algorithm's treatment recommendation is not ideal or suboptimal for the patient's condition. It may have considered some relevant clinical data but failed to account for other important factors. |
| 3 - Average (Neutrality) | The algorithm's treatment recommendation is generally appropriate for the patient's condition. It has considered most of the relevant clinical data and has arrived at a treatment decision that a physician might also recommend. |
| 4 - Above Average (Agreement) | The algorithm's treatment recommendation is well-suited for the patient's condition. It has taken into account all relevant clinical data and has arrived at a treatment decision that is aligned with an expert (breast oncologist/breast surgeon) recommendation. |
| 5 – Excellent (Total Agreement) | The algorithm's treatment recommendation is optimal for the patient's condition. It has thoroughly analyzed all relevant clinical data and provided a treatment decision that demonstrates a deep understanding of the patient's needs, and aligns with an expert (creast oncologist/breast surgeon) recommendation. |

**Supplementary Table 3. Explanation Scale:**

| Grade | Definition |
| --- | --- |
| 1 - Poor (Total Disagreement) | The algorithm's explanation for the clinical situation and its decision-making process is unclear, confusing, or incorrect. It may contain inaccuracies or irrelevant information. |
| 2 - Below Average (Disagreement) | The explanation provides some insight into the algorithm's decision-making process, but may lack clarity or coherence. It may contain inaccuracies or lack important details. |
| 3 - Average (Neutrality) | The explanation is generally clear and understandable, but could be improved in terms of detail or organization. |
| 4 - Above Average (Agreement) | The explanation effectively describes the patient’s clinical status and the algorithm's decision-making process. It is clear, coherent, and well-structured, with minimal room for improvement. |
| 5 – Excellent (Total Agreement) | The algorithm's explanation is accurate, and easy to understand. It thoroughly demonstrates the reasoning behind the decision-making process, leaving no room for improvement. |

**Supplementary Examples for queries:**

**Case #1:**

In this case there was complete agreement between the tumor board and the chatbot.

*Y.B:*

Hi, can I give you a patient story of breast cancer detected and you'll say what is the next step in her management? please decide if she needs surgery, what type of surgery, or whether she needs neo-adjuvant therapy before, or does she need further testing

*ChatGPT-3.5:*

Of course! I'd be happy to help. Please go ahead and give me the patient's story.

*Y.B:*

here is the story: 51 years old woman, no family hx, no hormonal meds. felt a mass in her left breast and did mammography and US. On US of her left breast in the outer-upper quadrant a 2 cm mass was detected. Then, a biopsy was performed to the mass and on pathology it was an IDC ER positive, PR negative, HER2 negative, KI high. Then she did an MRI for local staging - a solitary mass in her left breast was detected. She also did PET-CT with no evidence of distal metastases.

*ChatGPT-3.5:*

Based on the information provided, it appears that this patient has been diagnosed with Invasive Ductal Carcinoma (IDC) in her left breast.

Given the results of the pathology and imaging tests, the patient is most likely a candidate for surgery, as the mass is located in only one breast and there is no evidence of distant metastases.

For this type of breast cancer, the standard surgical options are either a lumpectomy (also known as a breast-conserving surgery) or a mastectomy (removal of the entire breast). In general, a lumpectomy is recommended for small, early-stage tumors, while a mastectomy may be recommended for larger tumors or for patients who have a higher risk of cancer recurrence.

In this case, given the size of the mass (2 cm), a lumpectomy with axillary lymph node dissection (removal of some of the lymph nodes in the underarm area to check for spread of cancer) would be the typical surgical approach. If a lumpectomy is performed, radiation therapy would usually be recommended to reduce the risk of recurrence.

It's worth noting that the patient's ER (estrogen receptor) positive, PR (progesterone receptor) negative, and HER2 (human epidermal growth factor receptor 2) negative status will play a role in determining her treatment options, as these receptors can be targeted with specific medications.

In conclusion, given the patient's diagnosis and test results, surgery, most likely a lumpectomy with axillary lymph node dissection, is the next recommended step in her management. After the surgery, the patient may also require additional treatment such as radiation therapy or systemic therapy (such as hormone therapy or chemotherapy) based on the results of the surgery and further testing.

**Case #2**:

In this case, there was a general consensus between the tumor board and ChatGPT-3.5 on the need for surgical treatment. While ChatGPT-3.5 agreed that a lumpectomy might be the most appropriate next step, it also advised discussing the option of mastectomy with the patient, considering she is a BRCA2 carrier and has previously undergone risk-reducing salpingo-oophorectomy. We found this perspective to be intriguing and potentially advantageous.

*Y.B:*

Hi, can I give you a patient story of breast cancer detected and you'll say what is the next step in her management? please decide if she needs surgery, what type of surgery, or whether she needs neo-adjuvant therapy before, or does she need further testing

*ChatGPT-3.5:*

Of course! I'd be happy to help you understand the next steps in the management of a patient with breast cancer. However, in order to make a proper recommendation, I would need more detailed information about the patient's medical history, their test results (such as biopsy results), and the stage and type of breast cancer they have been diagnosed with. Can you provide me with more information?

*Y.B:*

A 66 year old woman. BRCA2 carrier, generally health, previously underwent bilateral salpingo-oophrectomy. On recent breast MRI in her left breast, medial to the nipple in the front third of the breast a new mass measuring 0.8 cm was detected. No axillary lymphadenopathy was detected at MRI. The left breast mass was then biopsied under US guidance. The pathology from the biopsy was IDC grade 2, ER positive, PR positive, HER2 negative, KI67 20%.

*ChatGPT-3.5:*

Based on the information you've provided, the next step for this patient would likely involve a consultation with a breast surgeon and an oncologist to discuss her options for treatment.

Given that the patient is BRCA2 positive, has a new mass measuring 0.8 cm in her left breast, and the biopsy showed it to be IDC grade 2, ER positive, PR positive, and HER2 negative, the patient may be a candidate for surgery to remove the mass.

The most common surgical options for early stage breast cancer include:

Lumpectomy: removal of the tumor and some surrounding tissue

Mastectomy: removal of the entire breast

The patient's age, overall health, and history of bilateral salpingo-oophrectomy will also be taken into consideration when deciding on the best course of action.

It's important to note that every patient's case is unique and the best course of action will depend on a variety of factors, including the patient's medical history, the type and stage of their cancer, and their personal preferences.

It's recommended that the patient discuss all of her treatment options with her healthcare team and make a decision that is best for her individual situation.

*Y.B:*

Based on the guidelines alone, should she undergo lumpectomy and sentinal lymph node or mastectomy?

*ChatGPT-3.5:*

Based on the guidelines alone, it's difficult to determine with certainty whether a patient should undergo lumpectomy and sentinel lymph node biopsy or mastectomy. The final decision depends on a variety of factors, including the patient's individual medical history and the specifics of their diagnosis.

For this patient, given that she is BRCA2 positive and has a new mass measuring 0.8 cm in her left breast that is IDC grade 2, ER positive, PR positive, and HER2 negative, lumpectomy followed by sentinel lymph node biopsy would likely be the first line of treatment. However, the patient's age, overall health, and history of bilateral salpingo-oophrectomy will also be taken into consideration when deciding on the best course of action.

In some cases, a mastectomy may be recommended if the patient has a large tumor or if the cancer has spread to multiple areas of the breast.

It's important to note that the patient should discuss all of her treatment options with her healthcare team and make a decision that is best for her individual situation. The final decision should be based on a personalized approach that takes into account the patient's specific needs and preferences.

**Case #3:**

In the following case there was complete disagreement between the tumor board who recommended breast MRI as the next step, and the chatbot that recommended surgery. The tumor board team thought that re-evaluation of disease extent is warranted before re-excision.

*Y.B:*

Hi, can I give you a patient story of breast cancer detected and you'll say what is the next step in her management? please decide if she needs surgery, what type of surgery, or whether she needs neo-adjuvant therapy before, or does she need further testing

*ChatGPT-3.5:*

Sure! I'd be happy to help. Please provide the patient's story and I'll do my best to suggest a plan for her management.

*Y.B:*

54 years old,treated for hypertension, otherwise generally healthy. She has a family history of a maternal aunt with breast cancer at the age of 70+. She is still menstruating. She had no pregnancies and had 6 IVF cycles. She palpated a lump in her left breast. She underwent ultrasound. Her right breast was normal. In her left breast in the upper outer quadrant a lobular lump larger than 6 cm without a clear border was detected. On mammography a corresponding mass in her left breast was detected with no suspicious calcifications. The mass was biopsied under US guidance and the pathology result was fibroadenoma. She underwent lumpectomy to remove the large mass. The pathology result from the surgery was phyllodes tumor with mild atypia. The tissue received for pathology analysis was fragmented therefore assessment of surgical margin was not possible.

*ChatGPT-3.5:*

Based on the patient's story, the next step in her management would be to schedule her for re-excision or re-biopsy. Phyllodes tumor is a rare benign tumor but with mild atypia it is possible that some malignancy cells may be present. To confirm this, a complete assessment of the surgical margins is important. In cases where the tissue received for pathology analysis is fragmented, re-excision is recommended to obtain a more complete sample for accurate assessment of the surgical margins. If the re-excision is negative for malignancy, the patient may be followed up with regular imaging and clinical examinations to monitor for any changes. If malignancy is confirmed, additional treatment such as radiation therapy or mastectomy may be considered based on the patient's preference and oncologists' recommendations.

*Y.B:*

Should the patient undergo breast MRI before the surgery?

*ChatGPT-3.5:*

Breast MRI can be a useful tool in the evaluation of breast tumors, particularly in cases where mammography and ultrasound findings are equivocal or inconclusive. In this patient's case, since a mass was detected on ultrasound and mammography, and biopsy results confirmed the presence of a phyllodes tumor with mild atypia, a breast MRI may not be necessary before the re-excision surgery. However, this decision is best made in collaboration with the patient's healthcare team, taking into consideration the patient's specific clinical and imaging findings, as well as other factors such as the patient's age, family history, and general health status. If additional information is needed to further evaluate the lesion or surrounding tissues, a breast MRI may be recommended prior to surgery.
